# Delayed Severe Heart Failure Despite Successful Graves' Disease Management

**DOI:** 10.1002/ccr3.71693

**Published:** 2025-12-17

**Authors:** Hirotaka Nakashima, Takahiro Kamihara, Takuya Omura, Atsuya Shimizu

**Affiliations:** ^1^ Department of Cardiology National Center for Geriatrics and Gerontology Obu Japan; ^2^ Department of Internal Medicine Minami Seikyo Hospital Nagoya Japan; ^3^ Department of Metabolism National Center for Geriatrics and Gerontology Obu Japan

**Keywords:** ejection fraction, graves' disease, heart failure, management

## Abstract

Patients with Graves' disease may develop delayed heart failure with reduced ejection fraction even after achieving biochemical euthyroidism. Vigilance for cardiac complications and early referral to cardiology are essential.

## 1 Case Images

The estimated incidence of undiagnosed thyroid dysfunction is 3.8 [1], representing a significant patient demographic. Therefore, meticulous diagnosis and management of thyroid disorders are paramount in tertiary and primary care settings. This report presents a unique case of an older female who developed heart failure with reduced ejection fraction (HFrEF) accompanied by severe edema during the euthyroid phase several months following the initiation of treatment for Graves' disease.

A 63‐year‐old female with a medical history of Graves' disease presented with progressive dyspnea and exacerbating lower extremity edema. Four months prior, she was diagnosed with overt hyperthyroidism, characterized by suppressed thyroid‐stimulating hormone (TSH) and elevated free triiodothyronine (FT3) and free thyroxine (FT4), and was commenced on methimazole 15 mg/day. Although her thyroid function improved, she developed lower extremity edema 2 months later. Despite further amelioration of thyroid function and a reduction in methimazole dosage, she experienced significant weight gain (19 kg) and persistent edema. She discontinued methimazole 2 weeks before admission, yet her thyroid function remained within the normal range.

**FIGURE 1:**
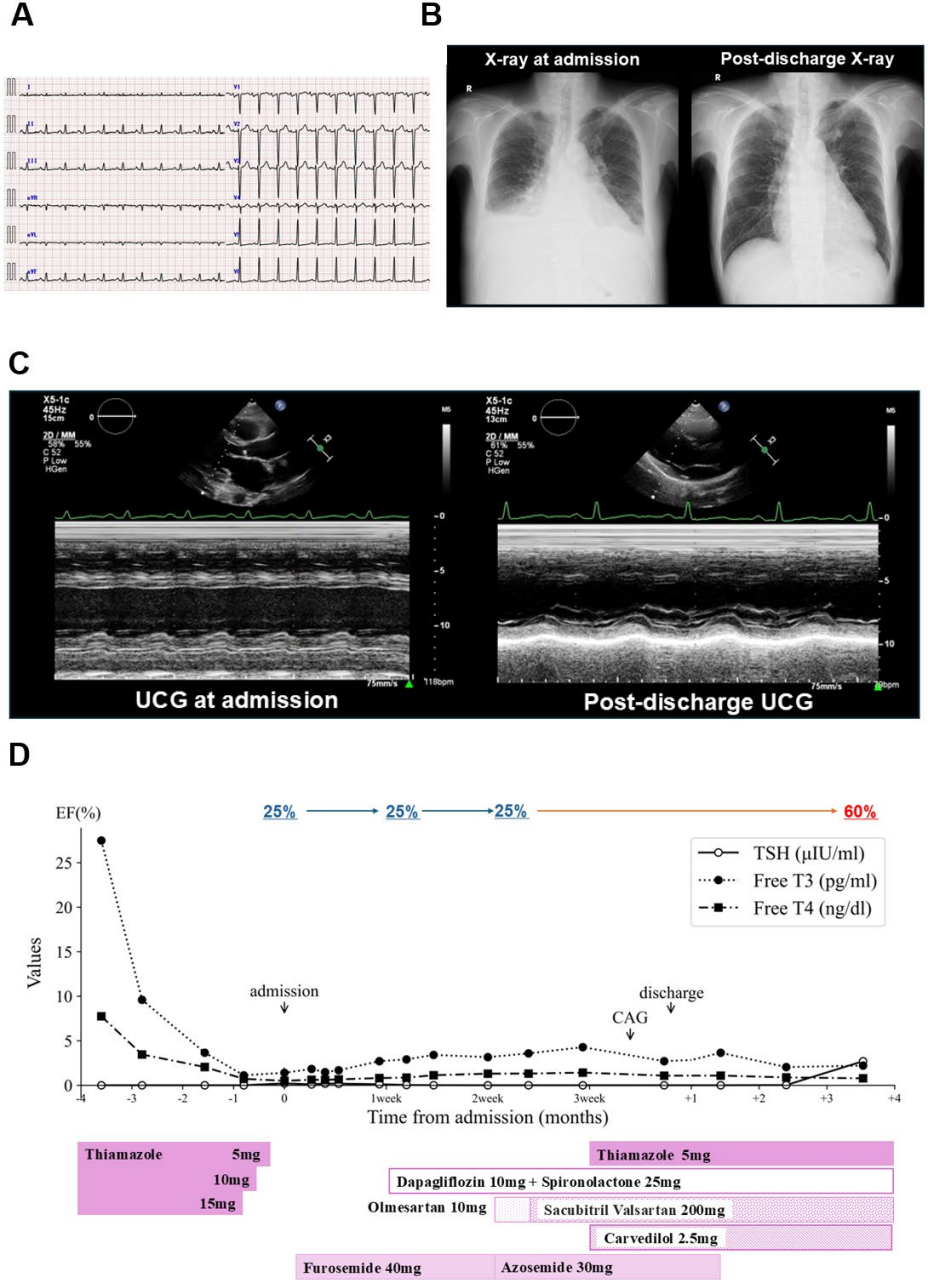
(A) Electrocardiogram demonstrating sinus tachycardia. No atrial fibrillation or ischemic changes were observed, which are commonly associated with hyperthyroidism. (B) Chest X‐ray findings at the initial visit and before discharge. The initial chest X‐ray showed significant pleural effusion (Left). The follow‐up chest X‐ray before discharge demonstrated a resolution of pleural effusion (Right). (C) Echocardiographic findings at the initial visit and after 3 months of treatment. Parasternal long‐axis M‐mode echocardiogram at the initial visit showed a severely depressed Left Ventricular Ejection Fraction (LVEF) = 25% (Left). A follow‐up echocardiogram using the same modality after 3 months demonstrated marked improvement in LVEF = 60% (Right). (D) Case chart. The patient's clinical course shows changes in thyroid hormone levels (FT3, FT4, TSH), treatment adjustments, and Left Ventricular Ejection Fraction over time. FT3, free triiodothyronine; FT4, free thyroxine; TSH, thyroid‐stimulating hormone.

Physical examination revealed coarse crackles bilaterally, a systolic murmur at the apex, and severe pitting edema. Laboratory findings showed elevated B‐type natriuretic peptide (BNP) and mild hypothyroidism (Table S1). Electrocardiography (ECG) revealed sinus tachycardia (Figure 1A), and chest X‐ray showed significant pleural effusion (Figure 1B). Transthoracic echocardiography demonstrated severe left ventricular systolic dysfunction with a left ventricular ejection fraction (LVEF) of 25% and diffuse hypokinesis (Figure 1C).

Guideline‐directed medical therapy for heart failure, including dapagliflozin, spironolactone, sacubitril/valsartan, and carvedilol, was initiated and titrated. Methimazole was restarted after stabilization of heart failure. Coronary angiography showed no significant stenosis, and myocardial biopsy was not performed per the patient's request. No atrial fibrillation was observed during continuous ECG monitoring.

Thyroid function gradually improved, but cardiac function did not recover during hospitalization. Symptoms and edema resolved, and the patient was discharged 1 month later. Follow‐up echocardiography 3 months post‐discharge showed improvement in LVEF to 60% (Figure 1C). The clinical course is summarized in Figure 1D.

Graves' disease is associated with cardiovascular complications, including atrial fibrillation and high‐output heart failure. However, HFrEF is rare [2]. Previous studies have identified prolonged hyperthyroidism and atrial fibrillation as risk factors for cardiomyopathy [3]. In this case, HFrEF developed despite achieving normalized thyroid function without atrial fibrillation. This case highlights the importance of considering HFrEF in patients with Graves' disease, even after achieving normalized thyroid function. The delayed or prolonged cardiac dysfunction is hypothesized to be related to several contributing factors, including residual myocardial remodeling, microvascular dysfunction, and autoimmune‐mediated cardiomyopathy, which may collectively impede sufficient recovery of cardiac function.

This case suggests that HFrEF, which carries a heightened risk of sudden cardiac death, may contribute to this increased mortality. This case underscores the importance of comprehensive management, including detailed history, physical examination, and additional investigations, to detect latent heart failure, even after achieving biochemical control of Graves' disease, for a wide range of physicians, including geriatricians.

## Author Contributions

**Hirotaka Nakashima:** writing – original draft, writing – review and editing. **Takahiro Kamihara:** formal analysis, funding acquisition, validation, writing – original draft, writing – review and editing. **Takuya Omura:** supervision, writing – review and editing. **Atsuya Shimizu:** writing – review and editing.

## Funding

This work was supported by the Japan Society for the Promotion of Science, 23K19602.

## Ethics Statement

This study was conducted in accordance with the Declaration of Helsinki with due consideration given to the patients. Ethics Committee of National Center for Geriatrics and Gerontology approved that the papers like this report do not warrant an ethical review.

## Consent

Written informed consent was obtained from the patient for the publication of this report in accordance with the journal's patient consent policy.

## Conflicts of Interest

The authors declare no conflicts of interest.

## Supporting information


**Table S1:** Blood test results at the initial visit.

## Data Availability

All data generated or analyzed during this study are included in this article and Supporting Information. Further inquiries can be directed to the corresponding author.
