# Supplementary material for: Delayed Severe Heart Failure Despite Successful Graves' Disease Management
Source: Clin Case Rep. 2025 Dec 17;13(12):e71693. doi: 10.1002/ccr3.71693 (PMC12710525; doi:10.1002/ccr3.71693)
Supplement: Supplementary file 1 — Table S1: Blood test results at the initial visit. [file CCR3-13-e71693-s001.docx]

**Supplemental Table 1. Blood test results at the initial visit.**

| Biochemistry |  |  | Complete blood count | |
| --- | --- | --- | --- | --- |
| TP (g/dL) | 6.7 |  | WBC (×102/μL) | 109 |
| Alb (g/dL) | 3.1 |  | RBC (×104/μL) | 498 |
| T-Bil (mg/dL) | 0.6 |  | Hb (g/dL) | 12.2 |
| AST (U/L) | 61 |  | Ht (%) | 39.6 |
| ALT (U/L) | 49 |  | Plt (×104/μL) | 25.9 |
| LDH (U/L) | 410 |  |  |  |
| CK (U/L) | 352 |  | Endocrinology |  |
| UA (mg/dL) | 6.5 |  | TSH (μIU/mL) | 0.21 |
| BUN (mg/dL) | 21 |  | FT3 (pg/mL) | 1.4 |
| Cre (mg/dL) | 0.92 |  | FT4 (ng/dL) | 0.5 |
| T-Cho (mg/dL) | 208 |  |  |  |
| LDL-C (,g/dL) | 126 |  | Cardiac Biomarker | |
| Na (mEq/L) | 141 |  | BNP (pg/mL) | 897 |
| K (mEq/L) | 4.4 |  |  |  |
| Cl (mEq/L) | 107 |  |  |  |
| CRP (mg/dL) | 0.06 |  |  |  |

TP, total protein; Alb, albumin; T-Bil, total bilirubin; AST, aspartate aminotransferase; ALT, alanine aminotransferase; LDH, lactate dehydrogenase; CK, creatine kinase; UA, uric acid; BUN, blood urea nitrogen; Cr, creatinine; T-Cho, total cholesterol; LDL-C, low-density lipoprotein cholesterol; CRP, C-reactive protein; WBC, white blood cell; RBC, red blood cell; Hb, hemoglobin; Ht, hematocrit; Plt, platelet; TSH, Thyroid stimulating hormone; FT3, Free triiodothyronine; FT4, Free thyroxine; BNP, Brain natriuretic peptide.
